# Supplementary material for: Whole-Genome Sequencing Revealed Local Genetic Differences and Multi-Layered Genetic Structure in Asian Brown Bear (Ursus arctos) Populations
Source: Genome Biol Evol. 2026 Aug 28;18(9):evag214. doi: 10.1093/gbe/evag214 (PMC13584206; doi:10.1093/gbe/evag214)
Supplement: Supplementary file 1 [file evag214_supplementary_data.zip › Supplementary_Figure.docx]

**Supplementary figure 1.** Genetic diversity per sample based on the heterozygosity (a) and fraction of genome in runs of homozygosity (b). The results for an individual from Etorofu (Iturup) Island, whose genetic diversity is low, are denoted with an asterisk. The bar colors represent correspond to the groups listed in Table S1: ancient brown bear, dark orange; ancient polar bear, light blue; cave bear, light orange; brown bears from West Asia, light green; brown bears from Central Asia, purple; brown bears from Central Russia, yellow; brown bears from East Asia, pink; brown bears from Europe, green; brown bears from Hokkaido, red; brown bears from North America, blue. Light colors (b) indicate the fraction of ROHs between 500 kb and 2 Mb in length, whereas dark colors (b) indicate the fraction of ROHs longer than 2 Mb.

**Supplementary figure 2.** PCA results of both modern and ancient samples. The results of the first and second principal components are presented in (a), and results of the third and fourth principal components are depicted in (b). The colors of dots in the PCA plots correspond to the worldwide map and the groups listed in Table 1. The shaded area in Figure S2a reflects the current habitat of the brown bear (McLellan et al. 2017).

**Supplementary figure 3.** PCA results of only modern samples. The results of the first and second principal components are presented in (a), and those of the third and fourth principal components are depicted in (b). The colors of dots in the PCA plots correspond to the worldwide map and the groups listed in Table 1. The shaded area in Figure S3a reflects the current habitat of the brown bear (McLellan et al. 2017).

**Supplementary figure 4.** Genetic structure results at *K*=2 (a), *K*=3 (b), *K*=4 (c), *K*=7 (d), *K*=8 (e), and EEMS (f) relative to sampling locations. The cross-validation (CV) error values for each number of *K* are shown in (g). The bar colors beneath the ADMIXTURE plot represent correspond to the groups listed in Table S1: brown bears from West Asia, light green; brown bears from Central Asia, purple; brown bears from Central Russia, yellow; brown bears from East Asia, pink; brown bears from Europe, green; brown bears from Hokkaido, red; brown bears from North America, blue. To avoid sampling bias, ADMIXTURE was conducted using only 20 individuals (Europe_191Y, Europe_OFS01, Europe_RF01, WAsia_Ge, WAsia_U12M, WAsia_U47M, Rus_235, Rus_U76, CAsia_Kazakhstan _U13M, CAsia_Tibet_U05M, CAsia_Tibet _U40, EAsia_U34, EAsia_UAA1, EAsia_ETF1, Hokkaido_421, Hokkaido_518, Hokkaido_819, NorthAmerica_ABC01, NorthAmerica_BB020, NorthAmerica_BB037). One sample (NorthAmerica_GT_1) was excluded from EEMS because we could not find detailed information regarding where it was captured. The shaded area in Figure S4a–e reflects the current habitat of the brown bear (McLellan et al. 2017).

**Supplementary figure 5.** Phylogenetic tree based on the mtDNA sequences and associated divergence times (in thousands of years ago). The number of individuals and total sites (n=118, 14,784 sites) are the same as in Figure 6a.

**Supplementary figure 6.** Phylogenetic tree based on the Y chromosome sequences and associated divergence times (in thousands of years ago). The number of individuals and total sites (n=30, 551,993 sites) are the same as in Figure 2.

**Supplementary figure 7.** PSMC results. Result of selected five individual (a), Central Asia (b), Etorofu(Iturup) Island and Hokkaido (c), West Asia and Europe (d), and East Asia (e). Line color on each PSMC result corresponded with dot color on the Eurasia continent map. The gray shaded area in the Eurasia continent map reflects the current habitat of the brown bear (McLellan et al. 2017).

**Supplementary figure 8.** TreeMix results. Number of migrations: *m* = 1 (a), *m* = 2 (b), *m* = 5 (c). The geographical names shown next to the circles indicate the location of each sample used in the analysis. Colors represent the groups listed in Table 1: ancient brown bear, dark orange; ancient polar bear, light blue; cave bear, light orange; brown bears from West Asia, light green; brown bears from Central Asia, purple; brown bears from Central Russia, yellow; brown bears from East Asia, pink; brown bears from Europe, green; brown bears from Hokkaido, red; brown bears from North America, blue. Shapes represent species: square, polar bear or cave bear; circle, brown bear.

**Supplementary figure 9.** Optimal number of migration edges determined using OptM. Mean and standard deviation (SD) for the composite likelihood L(m) per migration edges (a) and Δ*m*, in which a higher value indicates a more optimal migration edge (b). The numbers of migration edges pointed by arrows in Figure 10b represent the number of migrations shown in Figure 9.

**Supplementary figure 10.** Percentage of the admixture proportions from cave bears based on ƒ_4_ ratio. ƒ_4_ ratio (%) reflects the proportion of gene flow from cave bears to X, which represents a brown bear individual (a). The dot colors correspond to those on the world map (b). Colors represent the groups listed in Table 1: ancient brown bear, dark orange; ancient polar bear, light blue; cave bear, light orange; brown bears from West Asia, light green; brown bears from Central Asia, purple; brown bears from East Asia, pink; brown bears from Europe, green; brown bears from Hokkaido, red; brown bears from North America, blue. The gray shaded area in the worldwide map reflects the current habitat of the brown bear (McLellan et al. 2017). The other panels show the results for when the cave bear was HV74(taxa *kudarensis*) (c), GS136(taxa *ingressus*) (d), E_VD_1838(taxa *spelaeus*) (e), and WK01(taxa *eremus*). The tree topology shown in Figure S11a follows that presented to Figure 2 of Patterson et al. (2012).

**Supplementary Figure 11.** Migration rates through time between the polar bear (PB10) and brown bears from Hokkaido (Hokkaido_421; a), Etorofu (Iturup) Island (EAsia_ETF1; b), and North America (NorthAmerica_GP01; c), estimated using MSMC-IM. NorthAmerica_GP01 is from Glacier Park, Montana (Supplementary Table 1). The x-axis represents time (years ago), scaled assuming a generation time of 11 years (Benazzo et al. 2017; Endo et al. 2021). Black bold lines indicate the migration rate, and orange bold lines indicate the cumulative migration proportion. Thin lines represent bootstrap replicates.
